# Contribution of Arab researchers to ophthalmology: a bibliometric and comparative analysis

**DOI:** 10.1186/s40064-015-0806-0

**Published:** 2015-02-01

**Authors:** Waleed M Sweileh, Samah W Al-Jabi, Yousef I Shanti, Ansam F Sawalha, Sa’ed H Zyoud

**Affiliations:** Department of Pharmacology and Toxicology, College of Medicine and Health Sciences, An-Najah National University, Nablus, Palestine; Department of Clinical and Community Pharmacy, College of Medicine and Health Sciences, An-Najah National University, Nablus, Palestine; Department of Opthalmology, College of Medicine and Health Sciences, An-Najah University Hospital, Nablus, Palestine

**Keywords:** Bibliometric, Ophthalmology, Arab world, ISI Web of Science

## Abstract

Through history, Arabs and Muslims have made valuable contribution to medicine and science. The main objective of this study was to assess the contribution of Arab researchers to ophthalmology using bibliometric indicators. Published articles in “Ophthalmology” authored by Arab researchers were screened and analyzed using ISI Web of Science database. Worldwide research productivity in ophthalmology was 216,921 documents while that from Arab countries; Israel, Turkey and Iran were 2083, 2932, 3027 and 859 documents respectively. Those from Arab countries were published in 85 peer-reviewed ophthalmology related journals with 280 (13.44%) documents published in *Journal Francais d Ophtalmologie*. Among Arab countries, Kingdom of Saudi Arabia had the highest (828 (39.75%)) research output followed by Egypt (461 (22.13%)) and Tunisia 210 (10.08). Countries with highest collaboration with researchers in Arab world in ophthalmology research were USA; (397; 19.06%) followed by England (92; 4.42%) and Spain (91; 4.37%). The most research productive organization in Arab countries was King Khalid Eye Specialist Hospital (396; 19.01%). Ophthalmology articles authored or co-authored by an Arab researcher had a total citation f 21098 with an average citation of 10.13 per document and an *h*-index of 51. In conclusion, the present data show promising increase but relatively low ophthalmology research productivity from Arab countries. Wide variation in research productivity do exists. Compared with other non-Arab countries in the Middle East, Arab countries showed lesser ophthalmology research activity than Israel and Turkey but higher than that in Iran.

## Background

Ophthalmology is a branch of medicine that deals with health problems of the eye. Arab and Muslim scholar have made valuable contribution to modern medicine in general and ophthalmology in particular (Syed 2002; Huff 2003; Majeed 2005). For example Ibn al-Haytham (Alhazen), an Arab and Muslim scientist, wrote extensively on optics and the anatomy of the eye. Actually, Ibn al-Haytham is considered the one who made the first steps in the science of vision through his writings and explanations (Gorini 2003). Another Arab Muslim scientist is Ibn al-Nafis who lived in Damascus and wrote about medicine and eye diseases (Prioreschi 2006). In the past century, ophthalmology became an important medical, surgical, genetic and public health discipline. Furthermore, many medical institutions around the world became highly specialized in research, education and clinical practice of ophthalmology. Similarly, many new and potent ocular medications have been developed to overcome eye diseases including glaucoma, an important risk factor for blindness. Antibiotics have also made many eye infections easy to control and treat. Modern techniques and computerization have also helped detect and solve many visual problems. In the Arab world, medical education and clinical practice have witnessed a dramatic change especially in the past three decades. Many medical schools, hospitals, and specialized medical research centers have been established. However, it is believed that research in Arab countries in various medical fields is still lagging behind compared to non-Arab countries in the region (Al-Khader 2004; Benamer and Bakoush 2009; Bissar-Tadmouri and Tadmouri 2009; Farhat et al. 2013; Zyoud et al. 2014d). No data is available regarding the status and research output in ophthalmology from Arab countries. This specialty of medicine is of great importance to the Arab world because many risk factors for blindness, like diabetes mellitus, are prevalent in the Arab world. Actually, 6 out of the world’s top ten countries for highest prevalence of diabetes are in the Middle East (Whiting et al. 2011). Actually, many Arab researchers are aware of this and have launched peer reviewed journals dedicated for Ophthalmology to encourage Arab researchers in this field (e.g. Saudi Journal of Ophthalmology). Research in ophthalmology reflects excellence and quality of medical education, clinical practice, public health standards and public awareness related to eye and vision problems. One method to assess research activity in any country or region is to do bibliometric analysis which refers to the implementation of statistical methods for evaluating research productivity (Wallin 2005). Therefore, the objective of this study was to analyze research output by investigators in Arab countries in the field of ophthalmology. Bibliometric analysis of research activity as well as citation analysis of influential authors and researchers in ophthalmology has been investigated from different parts of the world (Davis and Wilson 2001, 2003; Ohba 2005; Ragghianti et al. 2006; Fan and McGhee 2008). However, up to the author’s best knowledge, none has been published from the Arab countries. It is believed that a bibliometric analysis in ophthalmology can lead to better preventive health policy and health services in the field of ophthalmology.

## Materials and methods

The data used in this study were based on the ISI Web of Science (WoS). All Arab countries, except Palestine, were used as country keys followed by “ophthalmology” as WoS category in advanced search engine of ISI WoS. Palestine was excluded from search keys because the Web of Science database does not recognize Palestine as an independent state yet. The time frame for the result was 1900 - 2012. The types of documents included were original research articles and review articles while other types were excluded.

Bibliometric indicators presented in the results include *h*-index which represents the number of citations received for each of the documents in descending order (Baldock et al. 2009; Schreiber 2013). Other indicators considered were top-ten ranked journals and the journal impact factor (IF) which was evaluated using the Journal Citation Report (JCR; Web of Knowledge) 2012 science edition by Thomson Reuters (New York, NY, USA). The *SCImago Journal Rank* (SJR) indicator was also included (Available at: http://www.scimagojr.com/SCImagoJournalRank.pdf). Scientific research output in the field of CAM was measured based on a methodology developed and used in previous bibliometric studies (Sweileh et al. 2014a; Sweileh et al. 2014d; Zyoud et al. 2014a, b; Zyoud et al. 2014d, e; Zyoud et al. 2014c). The collected data were used to generate the following information: (a) total and trends of contributions in ophthalmology research during all previous years up to the set date of data analysis (December 31th, 2012); (b) Arab countries research productivity and collaboration patterns; (c) journals in which Arab world researchers published; and (d) the citations received by the publications.

## Results

**Figure 1:**
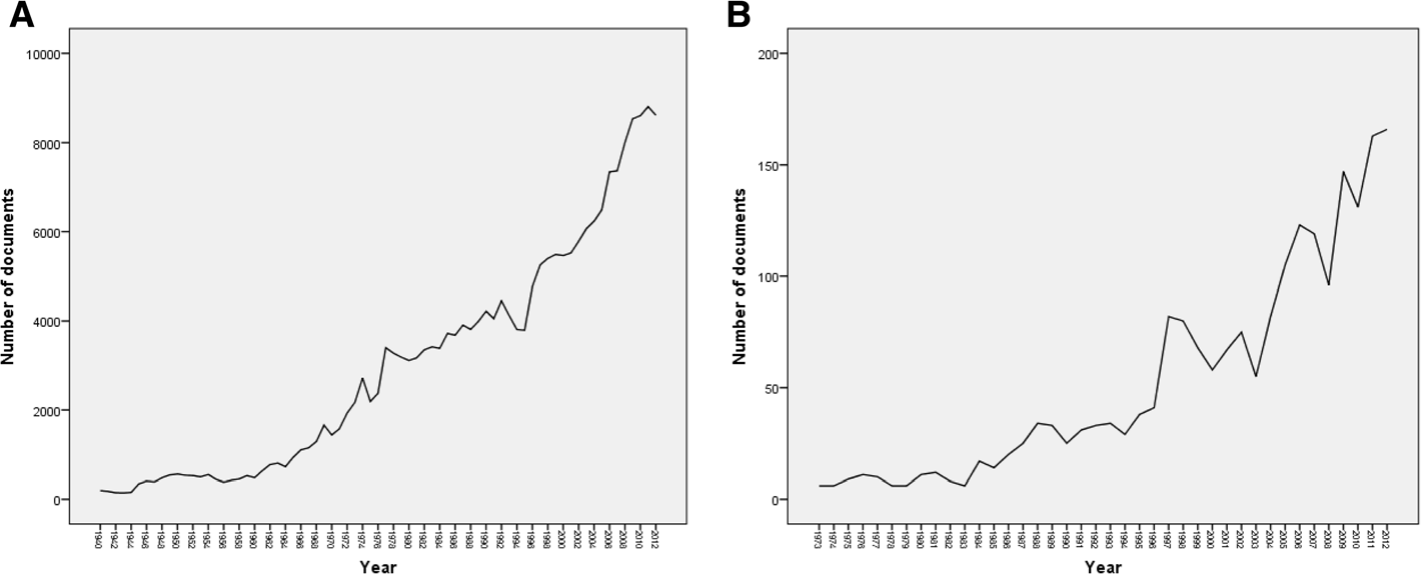
**Annual growth of ophthalmology research. A**. Worldwide. **B**. Arab world.

The total number of documents retrieved from ISI Web of Science using “Ophthalmology” subject category without specifying the name of any country was 216,921. This number represents the global research productivity (original research articles and reviews) in ophthalmology subject up to year 2012. When the same methodology was applied using the list of the 21 Arab countries, 2083 documents (2035 original articles and 48 review articles) in ophthalmology were retrieved. Therefore, research output in ophthalmology from Arab countries represents 0.96% of the global research productivity in ophthalmology. The annual number of documents published from Arab countries indicated that ophthalmology research output remained low until mid-1990s (Figure 1A and B). More than 50% of documents were published after the year 2005. The language of most documents was English (1780; 85.45%) followed by French (286; 13.73%), and German (17; 0.82%) languages. The first ophthalmology article from Arab countries was published in 1933 in *Archives of Ophthalmology (*currently named as *JAMA ophthalmology)* from Egypt with the following title “Trachoma in Egypt” (Olitsky and Tyler 1933).

The retrieved documents were published in 85 peer-reviewed ophthalmology related journals. Two hundred and eighty documents (13.44%) were published in *Journal Francais d Ophtalmologie* which started in 1978. Table 1 lists the top 10 journals in which documents in the field of ophthalmology were published from the Arab countries. Of the 2083 ophthalmology documents, there were 291 (13.97%) documents in surgery, 128 (6.15%) in pediatric and 60 (2.88%) in biochemistry/ molecular biology research area.

**Table 1:** **Top 10 journals in which ophthalmology documents from Arab countries were published**

**SCR**	**Journal name**	**Number of documents**	**IF** ^**a**^	**SJR** ^**b**^
		**N (100%) = 2083**		
**1** ^**st**^	*Journal Francais d Ophtalmologie*	280 (13.44)	0.438	0.23
**2** ^**nd**^	*British Journal of Ophthalmology*	149 (7.15)	2.725	1.7
**3** ^**rd**^	*Ophthalmology*	118 (5.67)	5.563	3.86
**4** ^**th**^	*Journal of Cataract and Refractive Surgery*	102 (4.90)	2.527	1.64
**5** ^**th**^	*American Journal of Ophthalmology*	100 (4.80)	3.631	2.96
**6** ^**th**^	*Journal of Refractive Surgery*	94 (4.51)	2.474	1.93
**7** ^**th**^	*European Journal of Ophthalmology*	75 (3.60)	0.912	0.67
**8** ^**th**^	*Journal of AAPOS*	70 (3.36)	0.731	0.59
**9** ^**th**^	*Eye*	69 (3.31)	1.818	1.18
**10** ^**th**^	*Molecular Vision*	60 (2.88)	1.987	0.88

Abbreviations: SCR = Standard Competition Ranking; IF = impact factor; SJR = Scientific Journal Ranking.

^a^The impact factor was reported according to Institute for Scientific Information (ISI) journal citation reports (JCR) 2012.

^b^The Scientific Journal Ranking was reported according to “http://www.scimagojr.com/”.

When retrieved data were analyzed based on country contribution (Table 2), Kingdom of Saudi Arabia (KSA) had the highest (828 (39.75%)) research output followed by Egypt (461 (22.13%)) and Tunisia 210 (10.08). No data related to ophthalmology was found from Djibouti and Mauritania. Approximately 62% of ophthalmology research from Arab countries came from KSA and Egypt. Collaboration in ophthalmology research with non-Arab countries was evident. Countries whose researchers collaborated most with researchers in the Arab world include the United States of America (USA); (397; 19.06%) followed by England (92; 4.42%) and Spain (91; 4.37%); (Table 3). When data regarding collaboration was analyzed for the top three Arab countries, we found that countries whose researchers collaborated most with researchers from KSA include the USA (205; 9.8%), Belgium (36; 1.7%), UK (27; 1.3%); Canada (20; 1.0%), and Spain (12; 0.6%). Egypt researchers collaborated most with researchers from the USA (89; 3.3%), Spain (67; 3.2%), England (35; 1.7%), Switzerland (19; 0.9%), and Germany (17; 0.8%). In addition, Tunisia researchers collaborated most with researchers from the USA (17; 0.8%), France (16; 0.8%), India (4; 0.2%), Italy (4; 0.2%), and Japan (4; 0.2%). Of interest, the top productive institution was *King Khalid Eye Specialist Hospital* in KSA (47; 11.03%). Five of the top 10 productive institutions were based in KSA while 4 were based in Egypt (Table 4).

**Table 2:** **Contribution of each Arab country in ophthalmology research output**

**Country**	**Number of documents**
	N (%) = 2083 (100%)*
Saudi Arabia	828 (39.75)
Egypt	461 (22.13)
Tunisia	210 (10.08)
Lebanon	184 (8.83)
Morocco	133 (6.39)
Oman	98 (4.71)
Kuwait	56 (2.69)
United Arab Emirates	49 (2.35)
Jordan	47 (2.26)
Algeria	22 (1.06)
Syria	16 (0.77)
Qatar	15 (0.72)
Sudan	11 (0.53)
Bahrain	7 (0.34)
Libya	7 (0.34)
Iraq	6 (0.29)
Yemen	5 (0.24)
Comoros	2 (0.1)
Somalia	2 (0.1)
Mauritania	0
Djibouti	0

*Total exceeds 100% because of overlap in some documents among more than one Arab country.

**Table 3:** **Top 10 countries whose researchers have collaborated with Arab researchers in publication of the 2083 documents in ophthalmology**

**SCR** ^**a**^	**Country**	**Number of documents**
		**N (%) = 2083 (100%)**
1^st^	United States of America	397 (19.06)
2^nd^	England	92 (4.42)
3^rd^	Spain	91 (4.37)
4^th^	Germany	52 (2.5)
5^th^	Belgium	50 (2.4)
6^th^	France	46 (2.21)
7^th^	Canada	45 (2.16)
8^th^	India	30 (1.44)
9^th^	Switzerland	27 (1.30)
10^th^	Japan	24 (1.15)

Abbreviations: SCR = Standard Competition Ranking.

**Table 4:** **Top 10 active organizations in the field of ophthalmology in Arab countries**

**SCR** ^**a**^	**Organization**	**Number of documents**	**Country**
		**N = 2083 (100%)**	
**1** ^**st**^	*King Khalid Eye Specialist Hospital*	430 (20.64)	KSA
**2** ^**nd**^	*King Saud University*	296 (14.21)	KSA
**3** ^**rd**^	*American University Beirut*	148 (7.11)	Lebanon
**4** ^**th**^	*Cairo University*	100 (4.8)	Egypt
**5** ^**th**^	*King Faisal Specialist Hospital Research Centre*	78 (3.75)	KSA
**6** ^**th**^	*Research Institute of Ophthalmology*	69 (3.31)	Egypt
**7** ^**th**^	*King Abdulaziz University Hospital*	64 (3.07)	KSA
**8** ^**th**^	*Ain Shams University*	50 (2.4)	Egypt
**9** ^**th**^	*University Alexandria*	47 (2.26)	Egypt
**10** ^**th**^	*The Eye Center*	42 (2.02)	KSA

Abbreviations: SCR = Standard Competition Ranking; KSA = Kingdom of Saudi Arabia.

**Figure 2:**
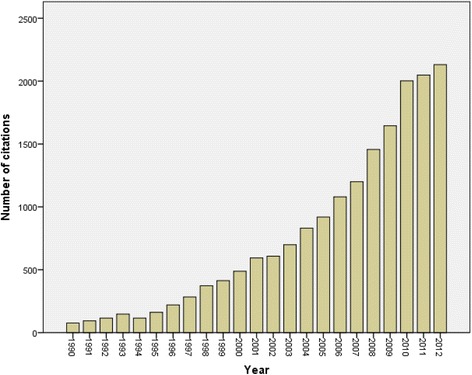
**Changes in total number of citation in the past 2 decades for ophthalmology documents published from Arab countries**

Table 5 shows the top 10 most active organizations in Arab countries in the field of Ophthalmology. The most research productive organization was King Khalid Eye Specialist Hospital (396; 19.01%). Nine out of top 10 active organizations are located in KSA or Egypt. The American University in Beirut was the only organization based outside the gulf and Egypt and was among the top 10 active organization. The total number of citations, at the time of data analysis (April 27th, 2014), was 21098 with an average citation of 10.13 per document. Of the 2083 documents considered for the h-index, 51 had been cited at least 51 times at the time of data analysis. Figure 2 shows the changes in the total number of citations in each year which reflects the changes in quality of publication in ophthalmology from Arab countries. Compared with other non-Arab countries in the Middle East, the research productivity from the Arab countries was lesser than that from Turkey (3027) and Israel (2932) and but higher than that from Iran (859). Table 5 shows a comparison between Arab countries and 3 non-Arab countries with regard to citation analysis for published ophthalmology documents.

**Table 5:** **Ophthalmology research output from Arab countries compared with that from Turkey, Israel and Iran**

**Variable**	**Arab countries**	**Israel**	**Iran**	**Turkey**
Number of results found	2083	2932	859	3027
Sum of the Times Cited	21098	39132	5282	24104
Sum of Times Cited without self-citations	19809	37066	4839	22136
Citing Articles	16135	30376	4216	17521
Citing Articles without self-citations	15460	29316	3961	16467
Average Citations per Item	10.13	13.35	6.15	7.96
*h*-index	51	70	31	46

## Discussion

The goal of the current study was to analyze the quality and quantity of ophthalmology research from Arab countries. It is true that few review articles about ophthalmology practice have been published from certain Arab countries in the past 2 decades (Tabbara and Blodi 1987; Wagoner and al-Rajhi 2001), however, no bibliometric analysis about ophthalmology research activity in Arab countries have been published. The importance of our study can be attributed to the following points: (1) ophthalmic diseases, including infectious diseases, and visual problems are common in Arab countries (Olitsky and Tyler 1933; Inhorn Millar and Lane 1988; Mansour et al. 1997; Tabbara 2001; Kotb et al. 2006; Saadi et al. 2007); (2) the huge urbanization and modernization, particularly in some Arab countries like Gulf states, need to be paralleled with a general increase in medical and ophthalmology research; (3) risk factors like poverty, poor hygiene, diabetes mellitus, hypertension, genetic diseases, poor nutrition are present in most Arab countries and finally (4) the advancement in the medical and surgical aspects of ophthalmology requires genuine attention from specialists and practitioners in Arab world to match their counterparts in non-Arab countries in Middle Eastern region.

Based on the results obtained in this study, research productivity from Arab countries in ophthalmology is relatively lagging behind. The rich heritage of Arabs in the field of vision and ophthalmology should give Arab nations a huge momentum in this field. With a total population of approximately 400 million, the number of documents in ophthalmology published from Arab countries requires a thoughtful analysis. Several potential reasons could be cited here. For example, the lack of specialists in this field might be a major contributor for the relatively low research productivity. Up until recently, most medical students who wanted to specialize in ophthalmology have to travel abroad to get a specialization degree in ophthalmology. This affected the number of practicing clinical ophthalmologist in Arab countries and hence the research activity in this field. The second potential reason is the lack of governmental and non-governmental financial support for research in the field of ophthalmology and its public health and economic impact on Arab nations. No doubt that Arabs are lagging behind in most medical fields (Benamer and Bakoush 2009), yet, the consequences of ophthalmic problems such as blindness is an emotionally and economically devastating problem.

In the present study, 2083 documents were analyzed based on ISI web of science database. First of all, it should be stated that this number does not represent 100% of the ophthalmology research output from Arab countries. It should be noted that some journals are not indexed in ISI web of science and therefore publications from the Arab countries in non-indexed journals were not counted. For example, articles pertaining to ophthalmology that were published in *Saudi Journal of Ophthalmology* or *Journal of the Egyptian Ophthalmological Society* were not included in the analysis since these journals are not indexed in ISI Web of Science. Nevertheless, the results obtained represent the genuine research from Arab countries published in international journals with high reputation. This is actually a closer and more accurate presentation of the status of ophthalmology research in Arab countries. Furthermore, the data obtained in our study will serve as a baseline data for future evaluation and for comparative purposes with other medical fields or with ophthalmology research in non-Arab countries.

It was expected that ophthalmology research output was highest from KSA and Egypt. It is believed that these 2 countries had the highest research productivity in other medical fields (Shaban and Abu-Zidan 2003; Tadmouri and Bissar-Tadmouri 2003; Waast and Rossi 2010). This is explainable by the fact that KSA is a rich country and a lot of financial support has been pumped into medical research and services in KSA. In case of Egypt, being the largest in population size in Arab countries, contributes to the high research productivity compared with other Arab countries. Actually, the history of medicine and ophthalmology in Egypt goes back thousands of years ago (Maccallan 1925a, b, 1927; Bey 1929; Dollfus 1956; Bieganowski 2003; Eltoukhi 2003; Coster 2009). Furthermore, international collaboration in the field of ophthalmology research seems high in KSA and Egypt. International collaboration is an important method and mechanism to improve the quantity and quality of research productivity (Lee and Bozeman 2005; Abramo et al. 2009). Furthermore, the international collaboration can draw attention and increase the visibility of scientific publication from Arab countries (Basu and Kumar 2000). No doubt that collaboration with countries like USA, England and other European countries helps in capacity building in the field of ophthalmology.

Research output studies in the field of ophthalmology have been conducted in several parts of the world. Screening the literature shows at least 10 different bibliometric studies in the field of ophthalmology and ophthalmology-related topics (Davis and Wilson 2003; Ohba 2005; Guerin et al. 2009; Kumaragurupari et al. 2010; Waast and Rossi 2010; Liu et al. 2011; Wu et al. 2011; Xu et al. 2011; Zhao et al. 2011; Jiao et al. 2012; Dai et al. 2013; Huang et al. 2013; Liu et al. 2013). Of particular interest is an article published about worldwide geographical distribution of ophthalmology publications (Guerin et al. 2009). The authors concluded that the greatest gross contributors to ophthalmology publications were USA, England and Japan. However, population adjusted analysis showed that Singapore, Iceland, and Australia were the most prolific nations (Guerin et al. 2009). In the same article, Israel came as one of the top 10 prolific nations which support the findings of our study that showed high quantity and quality of ophthalmology research from Israel compared to Arabs, Turkey and Iran. Another interesting published document is one about contribution of Iran to ophthalmology publications (Katibeh et al. 2011). The authors of the study concluded that although contribution of Iranian scientists to the field is growing, the Iranian nation needs to do more to bridge the gap with other nations and to be more presented in the field worldwide (Katibeh et al. 2011).

Finally, our study has points of strengths and certain points of limitations. Our study is the first article to analyze the quantity and quality of research productivity in the field of ophthalmology from Arab region. Although the study showed that contribution of Arab nations to the field is low, the main purpose of this study is well delivered which is to direct attention and initiate discussion among ophthalmologists in this regard. Our study is not without limitations, most of which have been mentioned in other similar studies (Sweileh et al. 2014b; Sweileh et al. 2014c; Sweileh et al. 2014e, f). The use of ISI web of knowledge database made our study confined to documents published in journals indexed in ISI web of science. Furthermore, many articles in ophthalmology published from Arab countries might have been published in non-ophthalmology journals.

## Conclusion

The present data show promising increase but relatively low ophthalmology research productivity from Arab countries. Wide variation in research productivity among Arab countries do exists with KSA and Egypt being in the top while many other Arab countries had very low productivity. Compared with other non-Arab countries in the Middle East, Arab countries showed lesser quantity of ophthalmology publications than Israel and Turkey but higher than that in Iran. Arab countries, particularly those in the field of ophthalmology need to establish links and research collaboration with other well developed countries in the field.

## Abbreviations

SPSS: Statistical Package for Social Sciences
ISI: Institute for Scientific Information
KSA: Kingdom of Saudi Arabia
UAE: United Arab Emirates
SAR: Syrian Arab Republic
USA: United States of America
JCR: Journal Citation Report
IRB: Institutional Review Board
SCR: Standard Competition Ranking
IFs: Impact factors
SJR: Scientific Journal Ranking
